# Linguistic effects on news headline success: Evidence from thousands of online field experiments (Registered Report Protocol)

**DOI:** 10.1371/journal.pone.0257091

**Published:** 2021-09-15

**Authors:** Kristina Gligorić, George Lifchits, Robert West, Ashton Anderson

**Affiliations:** 1 School of Computer and Communication Sciences, Ecole polytechnique fédérale de Lausanne (EPFL), Lausanne, Switzerland; 2 Department of Computer Science, University of Toronto, Toronto, Canada; Royal Holloway University of London, UNITED KINGDOM

## Abstract

What makes written text appealing? In this registered report protocol, we propose to study the linguistic characteristics of news headline success using a large-scale dataset of field experiments (A/B tests) conducted on the popular website Upworthy comparing multiple headline variants for the same news articles. This unique setup allows us to control for factors that can have crucial confounding effects on headline success. Based on prior literature and a pilot partition of the data, we formulate hypotheses about the linguistic features that are associated with statistically superior headlines. We will test our hypotheses on a much larger partition of the data that will become available after the publication of this registered report protocol. Our results will contribute to resolving competing hypotheses about the linguistic features that affect the success of text and will provide avenues for research into the psychological mechanisms that are activated by those features.

## Introduction

The spread of news and other important information has changed significantly in the age of online social media. As readers increasingly obtain their news over social media [1,2], publishers must engage their readers with individual articles rather than complete newspapers, in what has been dubbed the “unbundling of journalism” [3,4]. Since the same phenomenon gives readers the freedom to obtain news from many sources, publishers are engaged in fierce competition for their readers’ attention [4]. Moreover, the nature of online distribution has allowed news organizations to measure engagement at an unprecedented level of granularity and to experiment with distribution methods at a low cost [4–7]. For news publishers, these technological changes have emphasized the importance of crafting an engaging first impression, and have provided the technical infrastructure to conduct rigorous optimization tools for doing so. Publishers, however, ultimately have a limited ability to guarantee the success of their own output and must focus on ensuring that their content is of high quality. For news headlines, this implies developing knowledge of the linguistic predictors of textual success.

Understanding the linguistic features that promote information sharing has broad implications. It can shed light on what compels people to view and share online content. Knowledge of the linguistic features that cause success could be used by benevolent and malevolent actors alike. Benevolent actors might aim to optimize linguistic features in order to maximize engagement in high-stakes contexts such as public health messaging [8,9]. Malevolent actors, on the other hand, might aim to design clickbait [10] and to optimize the linguistic features by tapping into curiosity and interests as the driving mechanisms [11–13]. There has been substantial scrutiny of the predictors of success in various domains of text production. On our present focus of news, Berger & Milkman [14] studied the characteristics of *New York Times* articles that were heavily shared, identifying that articles that express positive or high-arousal emotions have a higher likelihood of becoming popular. A broad literature focuses on predicting success in news by various means [15–21], although much of this literature prioritizes prediction accuracy above the interpretation of features. The linguistic predictors of success have, however, been studied in other domains. For example, in online social media, Tan et al. [22] discovered several linguistic characteristics of tweets that outperformed closely matched alternatives in an observational study. Other studies on Twitter have investigated how sentiment [23], emotion [24], and length [25,26] affect tweet success. Aside from social media, other studies have used linguistic features to predict success in online communities [27], scientific abstracts [28], literature [29], and quotes [30].

Despite this existing literature, the relationship between linguistic traits and success remains unclear due to fundamental limitations. Broadly, prior work on success employs observational data, where the success outcome can be deeply confounded. Omitted-variable bias can drastically affect the modeled relationship between linguistic covariates and the success outcome to be predicted. For a domain such as news, a number of factors that are often correlated with success can be difficult to control for in observational studies. The time at which content is published can affect success due to concurrent events that create a demand for news or changes in audience size, so any comparison between items that occur at materially different points in time is generally invalid. The author of the content affects success both as a correlate of quality and as a source of social influence. Author skill (though difficult to observe) ought to affect quality, which itself brings success. More importantly, the “superstar phenomenon” [31] demonstrates that the audience for different authors can vary by orders of magnitude, while social influence can affect how an author’s content is received, independent of its quality [32]. In a study of Twitter popularity, it was shown that a model including only properties of the tweet author accounted for about half of the optimal model’s predictive performance, while the other half was accounted for by the user’s past success [33]. Moreover, the content of the article is dependent on the topic it discusses, and different topics have differing audience sizes. The format in which the article is presented also affects its success. In online news, articles on a homepage are typically presented in a grid with a thumbnail image associated with the article. The appeal of the image may drive clicks more than the linguistic properties of the headline. The digital era has enabled some researchers to mine big data for natural experiments that convincingly account for some of these important confounds [22]. It is, however, difficult to fully control for these critically important confounds, which can fundamentally alter the conclusions of any observational study regarding the content-specific predictors of success.

In this report, we conduct an analysis of experimental field data that provides very strict controls as well as a number of other benefits. We focus on news headlines, which we argue can serve as a “model organism” in an endeavor to elicit the linguistic factors of textual success, as news headlines are specifically crafted to engage with readers at a psychological level. We study a large number of experiments that were conducted by Upworthy, a popular online news publisher, which provides a large sample to test tightly controlled covariates. Each data point of our analysis is a randomized controlled experiment, so all exogenous factors that affect success are strictly controlled for. The experiments cover a long span of time, such that any linguistic covariates of time will be averaged within the multi-year period. Since each experiment varies headline options for a fixed article, and contextual factors such as the thumbnail of the article and the rest of the homepage are also fixed, we can control for the endogenous confounds of author, content, and context. Finally, the scale of the website on which the experiments were conducted ensures that each experiment is conducted on a large sample and linguistic comparisons are performed with strict measures of statistical significance.

Our analysis is made possible by the Upworthy Research Archive [34], a large dataset of online headline variation experiments made available for research purposes. This rich field experiment data is made available through a partnership between academic researchers and former Upworthy staff. Upworthy was a highly influential online publisher in the U.S. media landscape between 2013 and 2015; in November 2013, Upworthy attracted 80 million unique viewers [35] and was referred to as “the fastest growing media company in the world” [36,37]. With the help of rigorous online experimentation, Upworthy and its contemporaries identified a linguistic style which has since been labeled “clickbait”, a recipe so successful at attracting online attention that in November 2016, Facebook publicly announced a modification of their content recommendation algorithms to curb the spread of clickbait [3,38].

**Fig 1:**
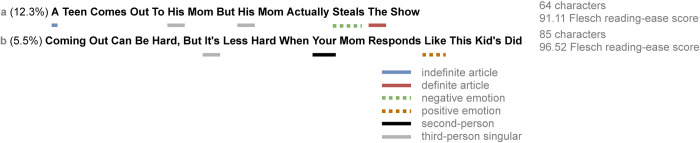
Example of a headline variation experiment pair and derived features. The *a* variant (top) had a higher clickthrough rate (12.3%) than the *b* variant (bottom; 5.5%). The *a* variant contains a definite and an indefinite article, a negative emotion word, and a third-person singular pronoun, whereas the *b* variant contains a positive emotion word, a third-person singular pronoun, and a second-person pronoun. Character count and Flesch reading-ease score are also shown.

The Upworthy Research Archive contains a total of 32,487 headline variation experiments conducted between January 2013 and April 2015 [39]. Each experiment is a comparison of several candidate headline variations authored for a target article, as illustrated in Fig 1. Visitors to the homepage of the Upworthy website were shown a selection of articles to view, and for the article that was the subject of any particular experiment, visitors were randomly assigned to see one variant of the headline. Every time the headline variant was shown to a visitor, as well as each time a visitor clicked on that headline variant, the event was logged. This design is referred to as an A/B test [6,7], and its randomized controlled nature allows the experimenter to identify which of several variants has a superior causal effect on clickthrough to its alternatives.

The A/B tests were conducted such that, when the Upworthy homepage was loaded, one article showcased on the homepage was selected for an experiment, with its headline and image varied across experimental conditions. According to former Upworthy engineers, in each experiment only one article on the homepage was varied [38]. Image contents are unavailable in the experimental data, but a unique image ID used for each variation is available, allowing researchers to ensure that the image is held fixed in headline comparisons.

Aside from the unprecedented scale and nature of the dataset, the two-stage process by which this data is made public also follows a novel paradigm. Out of 32,487 total experiments, a time-stratified subset of 4,873 experiments was made available for pilot research. In this registered report, we used this subset as pilot data in order to develop our analysis methodology, form hypotheses, posit the direction and size of the effects, and write the present registered report protocol. The remainder of the data will be available to researchers whose registered report protocol has been peer-reviewed and accepted. This release process ensures that all proposed hypotheses will be rigorously tested on a large, unseen dataset without publication bias. The unprecedented scale and experimental nature of the data, together with the scientific rigor of the release process, create an opportunity for conducting valuable confirmatory analysis.

Within the above-described experimental framework, we will test hypotheses that we developed based on an exploratory analysis of the pilot dataset or that have been proposed as important factors of success by prior literature. Our pre-registered analyses will assess eight specific hypotheses. The hypotheses, with the respective sampling plan, analysis plan, and the planned interpretation of the outcomes are summarized in the Design Table (Table 1).

**Table 1:** Design table.

Question	Hypo-thesis	Sampling Plan	Analysis plan	Interpretation given to different outcomes
The more successful headline in a controlled pair can be predicted at a statistically significant level based on linguistic features.	H1	See †	Evaluate test accuracy on the Confirmatory Dataset. The regression weights will be the same as those obtained in the Pilot data regression	Null hypothesis: classification accuracy is not significantly better than a majority vote classifier. Accept H1 if the Pilot data regression obtains significantly better accuracy on the Confirmatory Dataset headline pairs with α = 0.01.
The presence of positive-emotion words is negatively associated with headline success.	H2a	See ‡	Examine the regression coefficient “positive emotion”	Accept H2a if the coefficient is negative and statistically significant with α = 0.01, reject otherwise.
The presence of negative-emotion words is positively associated with headline success.	H2b	‡	Examine the regression coefficient “negative emotion”	Accept H2b if the coefficient is positive and statistically significant with α = 0.01, reject otherwise.
Length is positively associated with headline success.	H3	‡	Examine the regression coefficient “number of characters”	Accept H3 if the coefficient is positive and statistically significant with α = 0.01, reject otherwise.
Higher readability is negatively associated with headline success.	H4	‡	Examine the regression coefficient “Flesch reading-ease score”	Accept H4 if the coefficient is negative and statistically significant with α = 0.01, reject otherwise.
Generality (the use of indefinite articles) is positively associated with headline success.	H5a	‡	Examine the regression coefficient “indefinite article”	Accept H5a if the coefficient is positive and statistically significant with α = 0.01, reject otherwise.
Specificity (the use of the definite article) is negatively associated with headline success.	H5b	‡	Examine the regression coefficient “definite article”	Accept H5b if the coefficient is negative and statistically significant with α = 0.01, reject otherwise.
The use of first-person singular pronouns (referring to the author) is positively associated with headline success.	H6a	‡	Examine the regression coefficient “first-person singular”	Accept H6a if the coefficient is positive and statistically significant with α = 0.01, reject otherwise.
The use of first-person plural pronouns (referring to the author and the reader) is negatively associated with headline success.	H6b	‡	Examine the regression coefficient “first-person plural”	Accept H6b if the coefficient is negative and statistically significant with α = 0.01, reject otherwise.
The use of second-person pronouns is positively associated with headline success.	H7	‡	Examine the regression coefficient “second-person”	Accept H7 if the coefficient is positive and statistically significant with α = 0.01, reject otherwise.
The use of third-person singular pronouns is positively associated with headline success.	H8a	‡	Examine the regression coefficient “third-person singular”	Accept H8a if the coefficient is positive and statistically significant with α = 0.01, reject otherwise.
The use of third-person plural pronouns is positively associated with headline success.	H8b	‡	Examine the regression coefficient “third-person plural”	Accept H8b if the coefficient is positive and statistically significant with α = 0.01, reject otherwise.

† Performed on all headline pairs in the Confirmatory Dataset obtained following analysis pipeline described in Methods.

‡ All headline pairs obtained following analysis pipeline will be scored on the features, and regression analysis will be performed on all pairs.

### Predictability

The first hypothesis validates the basic premise of our analyses: Is it possible to attribute headline success to the linguistic features of headlines? Success can be the consequence of many complex factors at play, many of which are not observable or subject to unpredictable external shocks [40,41]. It has been shown that even a fully-described complex system can be so prone to the accumulated effects of random behavior that reasonable predictability is impossible [32,33,42]. It is therefore not *a priori* clear that the success of content in complex sociotechnical systems can be predicted at all. However, the experimental nature of the Upworthy Research Archive data allows us to precisely control for time and topic, which accounts for complex social factors. Therefore, any differences in headline success should be almost entirely accounted for by the individual decisions of consumers, and by ensuring that paired comparisons have a sufficiently large sample size, the unobservable factors that affect individual-level decisions are averaged out. Our first analysis thus explicitly asks: Is there any systematic variation in the success of headlines that can be explained or predicted based on linguistic features?

*H1*: *The more successful headline in a controlled pair can be predicted at a statistically significant level based on linguistic features*.

Following this first high-level hypothesis, we next turn our attention to specific hypotheses about the individual linguistic factors of headline success and discuss literature which supports them.

### Emotion

The use of emotional wording has been explored in several contexts. Prior work suggests that the use of emotional words increases sharing probability in several contexts, such as newspaper articles [14] and tweets [22,24]. Furthermore, the type of emotional reaction elicited by a text may affect its success. Past work supports conflicting views about broadly what kind of emotion is inherently more appealing to individuals. For example, the “Pollyanna hypothesis” [43] states that there is a human tendency to use positive language, a result that has been empirically verified across vast and diverse corpora [44]. In the online sphere, positive affect is more prevalent than negative affect on Twitter, indicating that people generally tend to tweet about happy things [45]. On the other hand, a general result suggests that bad events have greater power than good ones over a wide range of psychological scenarios, including that bad events elicit more information processing, stronger memory, and have more pronounced effects on impression formation [46]. For social media, it has been shown that positive content may receive more popularity than negative content [23,27], but negative messages spread faster [23], and negative words are more likely to be perceived as relevant to success [26]. Based on these latter results and our findings from pilot data, we form the following two hypotheses:

*H2a*: *The presence of positive-emotion words is negatively associated with headline success*.
*H2b*: *The presence of negative-emotion words is positively associated with headline success*.

### Length

The appeal of a headline may be associated with its length via competing factors. A Gricean maxim of cooperative communication emphasizes that the *quantity* of transmitted information should be sufficient to be informative, but only as much as required [47]. Meanwhile, the maxim of *relation* may favor brevity, introducing a tension between being informative and being concise [48,49]. There are reasons why shorter headlines may be expected to perform better. Shorter posts were found to be more successful on Twitter, with length constraints improving tweet quality [25], and by shortening original tweets to various lengths, Gligorić et al. [26] found that tweets which are up to 30–40% shorter than their longer original versions are more likely to be judged as successful. Accordingly, in a study of phrases of text being repeated in various online sources, Simmons et al. [50] found that shorter phrases were used more often. On the other hand, a longer headline may contain more information, with a higher probability of engaging the reader [22], a hypothesis that is indeed supported by our analysis of pilot data:

*H3*: *Length is positively associated with headline success*.

### Readability

Highly readable text may be more sympathetic to the reader, while less readable text may provide more information. A matched observational study of topic- and author-controlled tweets revealed that tweets with higher readability are more likely to be successful [22]. However, the linguistic style of text posted on Twitter is substantially different from text present in other corpora of other online content such as online blogs [51] or the news headlines studied here. In particular, Hu et al. [45] described how stylistic features correlated with readability vary significantly across media. In a study of successful literary works, Ashok et al. [29] found that readability was negatively associated with success. A proposed explanation is that great literature demonstrates high conceptual complexity, which in turn demands lower readability. For the present domain of news headlines, our preliminary analysis of pilot data yielded no significant effect. Aligned with the work of Ashok et al. we therefore state the following hypothesis:

*H4*: *Higher readability is negatively associated with headline success*.

We measure readability with the Flesch reading ease score [52], which decreases as either words per sentence increase or syllables per word increase. Thus, higher values imply that the text is more readable.

### Generality

Broadly speaking, the use of indefinite articles (“a”, “an”) can signal generality in the subject discussed [30], whereas the definite article (“the”) typically makes reference to something specific and unique [53]. Regarding the appeal of text, Danescu-Niculescu-Mizil et al. [30] found that the usage of general language made movie quotes more likely to be remembered. Similarly, Tan et al. [22] found in their study of topic- and author-controlled tweets that the inclusion of indefinite articles had a positive effect on tweet success. Our pilot analysis yielded no evidence that the usage of these articles has a significant effect on headline success. Based on the existing work, we thus form the following hypotheses:

*H5a*: *Generality (the use of indefinite articles) is positively associated with headline success*.
*H5b*: *Specificity (the use of the definite article) is negatively associated with headline success*.

### Pronouns

The use of pronouns in a headline can indicate whether the headline is inclusive of the reader, the author, or refers to a third party. Different pronouns can significantly alter the tone of the headline and certain pronouns may be broadly preferable to readers in general. For instance, Ashok et al. [29] found that pronouns were associated with highly successful books. We consider first-, second-, and third-person pronouns separately. First-person pronouns in particular have been found to contribute to success in scientific abstracts [28], but were not found to correlate with success in tweets [22]. In our pilot analyses, there was a significant positive effect of the inclusion of first-person singular pronouns, which refer to the author, but a negative and non-significant effect of the inclusion of first-person plural pronouns, which refer to a collective that may include both the author and the reader.

*H6a*: *The use of first-person singular pronouns is positively associated with headline success*.
*H6b*: *The use of first-person plural pronouns is negatively associated with headline success*.

Second-person pronouns refer directly to the reader (i.e., “you”). A prediction study of news headline popularity found that second-person pronouns are associated with more popular headlines [20]. A study of the success of songs found that the use of second-person pronouns is empirically correlated with song success and has a positive causal effect on people liking a song [54]. Our pilot analysis yielded a non-significant positive effect of second-person pronouns on headline success. Aligned with previous work, we thus form the following hypothesis:

*H7*: *The use of second-person pronouns is positively associated with headline success*.

Third-person pronouns were found to have a positive effect on tweet success [22]; they were, however, not found to be associated with popularity in a study of news headlines [20]. To the best of our knowledge, no prior work has found a significant distinction between third-person singular and plural pronouns. Our analysis of pilot data found that third-person *singular* pronouns (i.e., “she”, “his”) were positively associated with more engaging headlines, whereas third-person *plural* pronouns (i.e., “they”, “theirs”) were positively and non-significantly associated with headline success, so we hypothesize:

*H8a*: *The use of third-person singular pronouns is positively associated with headline success*.
*H8b*: *The use of third-person plural pronouns is positively associated with headline success*.

## Methods

### The use of exploratory and confirmatory datasets

At a high level, our work involves two analyses that hinge on one logistic regression model: the first analysis aims to determine whether the model has meaningful out-of-sample predictive accuracy, whereas the second analysis interprets the regression coefficients to assess factors that are associated with headline performance. The release schedule of the Upworthy Research Archive is intended to prevent scientific methodological errors that threaten the validity of hypotheses formed based on the data (such as p-hacking or cherry-picking subsets of the data). In this section we describe how our analysis makes use of each portion of the dataset.

Note that we do not propose any exploratory analyses in this registered report protocol. Our use of the phrase “Exploratory Dataset” follows terminology from the Upworthy Research Archive team, and simply refers to the initial stage of the Upworthy Research Archive data release. We treat the Exploratory Dataset as the pilot data based on which we designed our methodology and formed our hypotheses.

#### H1: Evaluation of predictability of headline success

A common issue in statistical learning is *overfitting*, in which an estimator exploits associations between predictors and the outcome that are idiosyncratic to the training data. Since there is a high probability of random associations occurring, an overfitted estimator will have high accuracy within the training sample. However, the goal of most statistical learning applications is *generalization*, or finding rules that yield good predictive performance on unseen data. Techniques such as cross-validation are designed to estimate generalization accuracy [55], but the most reliable assessment uses a large portion of the dataset that was never used in the training process. We therefore evaluate H1 by testing the predictive performance of the logistic regression model trained on the *Exploratory Dataset*, using the Confirmatory Dataset as a large held-out testing dataset.

#### H2-H8: Evaluation of linguistic hypotheses

Our second analysis involves the interpretation of logistic regression coefficients to probe the specific meaning of effects that are observed in the data. For this analysis, it is necessary to fit a logistic regression to the Confirmatory Dataset and analyze the coefficients as described in the Design Table (Table 1).

### Design

We study the linguistic traits of headlines by examining how the presence of words increases the odds of a headline being considered better than its alternative. The unique randomized experimental setup in which the data was collected enables this research design by allowing one to disregard any omitted variables that are causally relevant to headline success. According to the Upworthy Research Archive team [38], the original assignment of readers to experimental conditions was random, and only the headline and article image was visible to Upworthy readers as part of any headline variation test. By controlling for headline variations with the same image and conducted within the same week, we ensure that any differences in headline success are fully accounted for by the differences in words used in the headlines themselves.

The Upworthy Research Archive consists of data on online headline variation experiments. Most of these experiments test several headline variations for any given article. Every time a specific headline variant is shown to a reader, it is counted as an *impression*, and when the headline variant is clicked it is counted as a *click*. The *clickthrough rate* for any particular headline variant is defined as *clicks* divided by *impressions*. Experiments can vary other properties, but only the image ID, headline, and week during which the test was conducted is relevant to the impressions received on the Upworthy homepage [38].

Our research hypotheses require data about headlines that are better than a comparable alternative. We obtain pairs of headline variants by considering all possible pairs within each headline variation experiment, such that any headline pair under consideration has the same article ID and image ID, and was tested in the same week. Within each pair of headlines obtained this way, we define as “better” the headline with the higher clickthrough rate, and as “worse”, its counterpart.

#### Sampling plan

Groups of controlled experiments include varying numbers of comparable headlines that can be paired into comparison pairs. Within a group of controlled experiments, we consider every possible pair of comparison headlines. In case with more than *K* = 15 headline comparison pairs within an experiment, we randomly sample a subset of *K* = 15 comparison headline pairs. We have run the complete analysis pipeline for different values of *K*, obtaining the same effect directions and comparable effect sizes.

For many comparison pairs in the data, the better headline performed only marginally better than the worse headline, while for other pairs, there were only few impressions received by one headline variant. Within a group of controlled experiments, we perform a Pearson chi-squared test on the clickthrough rates for every possible pair. With each pairwise comparison in a headline experiment there is a probability of incorrectly rejecting the null hypothesis, so we apply the Bonferroni correction [56] with a family-wise error rate of α = 0.05. Note that in experiments testing more than two comparable headlines, each headline participates in a single comparison pair. Among such possible matchings of comparable headlines into distinct pairwise comparisons, we select the configuration with the lowest Bonferroni-corrected p-value.

When testing our hypotheses, the unit of analysis is a pair of comparable headlines, such that each headline among the analyzed set of pairs is unique. All hypotheses presented in this report were developed with the Exploratory Dataset release of the Upworthy Research Archive. With the Exploratory Dataset, we obtained 5,048 pairs of comparable headlines and performed an initial test of all hypotheses on this set of headline pairs. To support time-series research, both Confirmatory and Exploratory Datasets are a random sample of A/B tests, stratified by week number. The Confirmatory Dataset is expected to contain roughly four times the number of headline experiments. All hypotheses proposed in this report will be tested on the Confirmatory Dataset using the same Analysis Plan, but with a large sample of unseen data. All data pre-processing steps will be unchanged from what was developed on the Exploratory Dataset.

### Analysis plan

Since linguistic features are the primary object of study, our analysis focuses on counting words used in the headline pairs. We developed a dictionary of specific words which we considered for the set of pronoun categories, and used “a” and “an” for the *indefinite article* category (full dictionaries are available in Table 2). For the positive and negative emotion categories, we used Linguistic Inquiry and Word Count (LIWC) [57] to categorize individual words as possessing either positive or negative emotion. Finally, the *textstat* library for Python [58] is used to compute the Flesch reading-ease score [52] and the number of characters for each headline. This process is used to obtain a feature-vector encoding for each headline in the dataset. The process is depicted in Fig 1.

**Table 2:** Hypothesis word dictionaries.

Category	Words
first-person singular	i, i’d, i’ll, i’m, i’ve, id, im, ive, me, mine, my, myself
first-person plural	our, ours, ourselves, us, we, we’d, we’ll, we’re, we’ve
second-person	ya, you, you’d, you’ll, you’re, you’ve, youll, your, youre, yours, yourself, yourselves, youve
third-person singular	he, he’d, he’s, her, hers, herself, hes, him, himself, his, it, its, itself, she, she’ll, she’s, shes, themself
third-person plural	their, theirs, them, themselves, they, they’d, they’ll, they’ve, theyll, theyve
indefinite article	a, an
definite article	the

For each pair, we then compute feature vectors for the better and worse headlines. The feature encoding for each headline pair is the difference between the better headline’s features and the worse headline’s features. All linguistic features merely count the presence or absence of any words in the headlines: thus, for a headline pair, the linguistic feature is 1 if it only occurs in the better headline, -1 if it only occurs in the worse headline, and 0 if it occurs in neither or both headlines. The number of characters and the Flesch reading-ease score are real numbers, so the number-of-characters feature is 1 if the better headline is longer than the worse headline, -1 if the worse headline is longer than the better headline, and 0 if the two headlines are equally long; and the reading-ease feature is 1 if the better headline is easier to read than the worse headline, -1 if the worse headline is easier to read than the better headline, and 0 if the two headlines are equally readable. This constitutes the design matrix of predictors. In Table 3, we show the fraction of comparison pairs that differ in each of the features.

**Table 3:** Linguistic variables and the frequency in which they are manipulated among the comparison pairs.

Linguistic variable	Percentage of comparison pairs differing with respect to this linguistic variable
first-person singular	16.3%
first-person plural	14.7%
second-person	27.8%
third-person singular	31.0%
third-person plural	17.8%
indefinite article	37.4%
definite article	37.9%
positive emotion	38.4%
negative emotion	30.6%
number of characters	100%
Flesch reading-ease score	100%

An outcome vector of length *N* containing half zeros and half ones is generated and permuted; each row of features is then multiplied by -1 if the outcome is 0 and remains the same if the outcome is 1. This sets up a binary classification problem with perfectly balanced classes, such that either a majority-vote or random classifier would obtain 50% accuracy on this prediction task. For the linear model that we use in our analyses, a positive coefficient for a feature means that the feature was more prevalent in the better headline, whereas a negative coefficient means that the feature was more prevalent in the worse headline.

Each hypothesis in our report is defined by either a set of tokens, a deterministic rule for selecting tokens, or a deterministic function from headline text to output value. Our design matrix thus has one column to quantify each hypothesis. We fit a logistic regression on this design matrix and analyze the coefficients. As described in our Design Table (Table 1), each coefficient maps to a hypothesis. In the registered report to be compiled after analyzing the Confirmatory Dataset, we will say that a hypothesis is supported if its corresponding column in the regression has p < 0.01, although for our pilot analyses we considered p < 0.05 as preliminary evidence for the hypotheses.

### Results on pilot data

The Exploratory Dataset, which is a sample of 4,873 tests stratified by week [38]. As described above, we used the Exploratory Dataset to design research methods and develop the hypotheses presented in this report. A dataset of headline pairs is constructed, features are computed, and a logistic regression model is trained as described in the remainder of this section.

#### H1: Success prediction

Our first hypothesis posits that our model can predict headline success from linguistic features. Using our regression model, with 0.5 decision threshold on the predicted value, prediction accuracy on the pilot data was 55.23% (*Pearson χ*^2^(1) = 27.69, *P*<10^−6^, *odds ratio* = 1.23, 99% *CI* = [0.534, 0.570]), recall was 55.31%, precision was 55.22%. We conclude with the prediction that our model will identify the better headline from pairs in the Confirmatory Dataset significantly better compared to a random baseline.

#### H2-H8: Linguistic hypotheses

**Fig 2:**
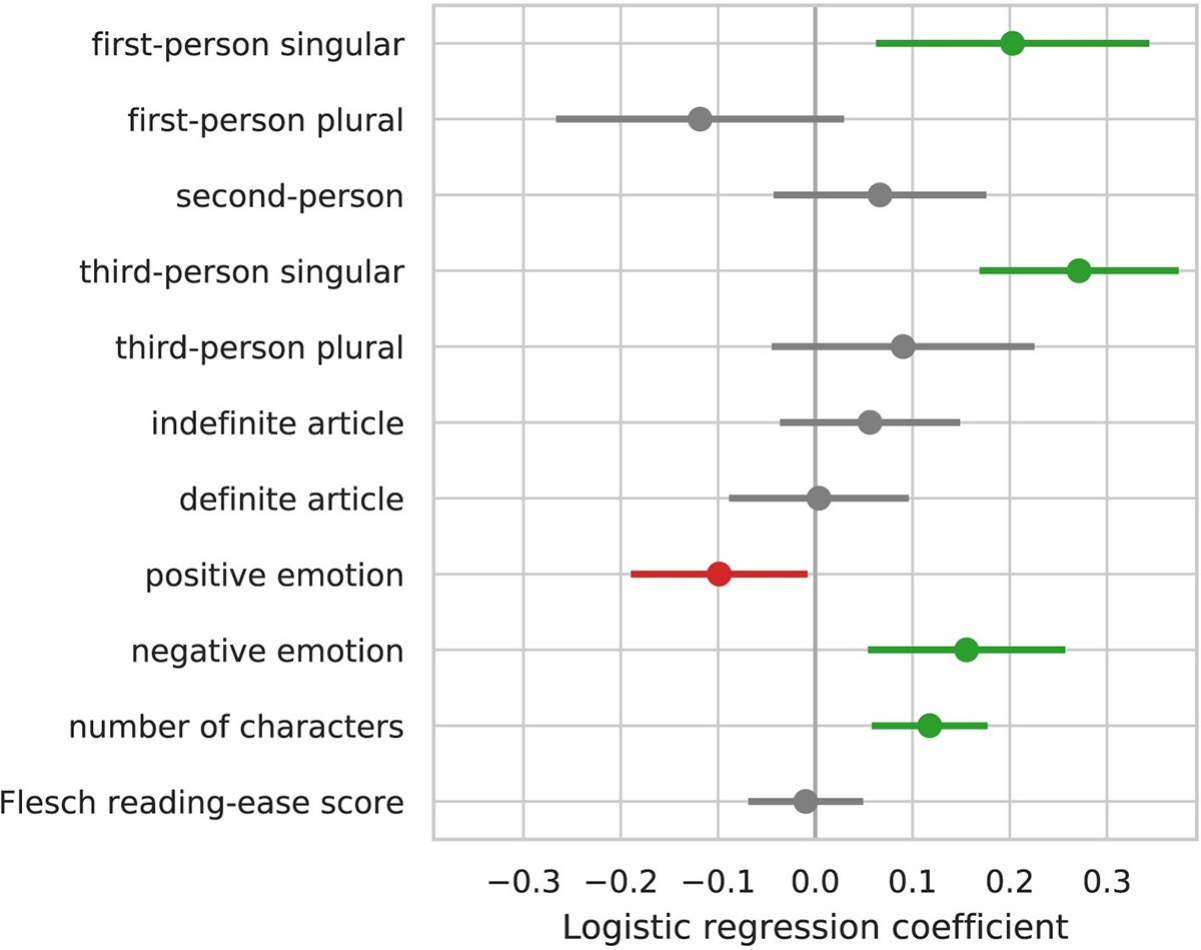
Main regression analysis on the Upworthy Archive Exploratory Dataset. Error bars show 95% confidence intervals on the logistic regression coefficients.

**T**he remaining hypotheses are assessed from inspecting the fitted regression coefficients (depicted in Fig 2; detailed statistics in Table 4). Our hypotheses (Design Table, Table 1), which posit the presence of an effect and its direction, are based on the Exploratory Dataset results obtained in Fig 2. Overall, we expect these results to generalize to the Confirmatory Dataset.

**Table 4:** Main analysis regression with Exploratory Dataset. Logit regression analysis for pilot data.

	Dep. Variable:		y	No. Observations:		5048
	Model:		Logit	Df Residuals:		5037
	Method:		MLE	Df Model:		10
	Date:		Sun, 27 Jun 2021	Pseudo R-squ.:		0.01102
	Time:		14:26:22	Log-Likelihood:		-3460.4
	Converged:		True	LL-Null:		-3499.0
	coef	std err	z	P>|z|	[0.025	0.975]
first-person singular	0.1878	0.072	2.613	0.009	0.047	0.329
first-person plural	-0.1228	0.075	-1.628	0.104	-0.271	0.025
second-person	0.0750	0.056	1.348	0.178	-0.034	0.184
third-person singular	0.2611	0.052	5.014	0.000	0.159	0.363
third-person plural	0.1047	0.069	1.525	0.127	-0.030	0.239
indefinite article	0.0486	0.047	1.026	0.305	-0.044	0.141
definite article	0.0047	0.047	0.099	0.921	-0.088	0.097
positive emotion	-0.0910	0.046	-1.969	0.049	-0.182	0.000
negative emotion	0.1540	0.052	2.986	0.003	0.053	0.255
number of characters	0.1209	0.030	3.981	0.000	0.061	0.180
Flesch reading-ease score	-0.0101	0.030	-0.337	0.736	-0.069	0.049
